# Substrate Stiffness and Oxygen as Regulators of Stem Cell Differentiation during Skeletal Tissue Regeneration: A Mechanobiological Model

**DOI:** 10.1371/journal.pone.0040737

**Published:** 2012-07-24

**Authors:** Darren Paul Burke, Daniel John Kelly

**Affiliations:** 1 Trinity Centre for Bioengineering, Trinity Biomedical Sciences Institute, Trinity College Dublin, Dublin, Ireland; 2 Department of Mechanical and Manufacturing Engineering, School of Engineering, Trinity College Dublin, Dublin, Ireland; The University of Akron, United States of America

## Abstract

Extrinsic mechanical signals have been implicated as key regulators of mesenchymal stem cell (MSC) differentiation. It has been possible to test different hypotheses for mechano-regulated MSC differentiation by attempting to simulate regenerative events such as bone fracture repair, where repeatable spatial and temporal patterns of tissue differentiation occur. More recently, *in vitro* studies have identified other environmental cues such as substrate stiffness and oxygen tension as key regulators of MSC differentiation; however it remains unclear if and how such cues determine stem cell fate *in vivo*. As part of this study, a computational model was developed to test the hypothesis that substrate stiffness and oxygen tension regulate stem cell differentiation during fracture healing. Rather than assuming mechanical signals act directly on stem cells to determine their differentiation pathway, it is postulated that they act indirectly to regulate angiogenesis and hence partially determine the local oxygen environment within a regenerating tissue. Chondrogenesis of MSCs was hypothesized to occur in low oxygen regions, while in well vascularised regions of the regenerating tissue a soft local substrate was hypothesised to facilitate adipogenesis while a stiff substrate facilitated osteogenesis. Predictions from the model were compared to both experimental data and to predictions of a well established computational mechanobiological model where tissue differentiation is assumed to be regulated directly by the local mechanical environment. The model predicted all the major events of fracture repair, including cartilaginous bridging, endosteal and periosteal bony bridging and bone remodelling. It therefore provides support for the hypothesis that substrate stiffness and oxygen play a key role in regulating MSC fate during regenerative events such as fracture healing.

## Introduction

The analysis of regenerative events such as fracture healing in long bones has led to the development of a number of theories on how the local mechanical environment regulates stem cell differentiation. Over 50 years ago, Pauwels hypothesised that distortional shear stress is a specific stimulus for collagen fibres and that cartilage formation is induced by a compressive stress stimulus [1]. Bone formation, it was argued, could only occur after soft tissues had ensured sufficient stabilisation of the callus. Inspired by Pauwels' initial hypothesis, a number of investigators have proposed alternative mechanical stimuli as regulators of stem cell fate. Using computational tools such as finite element analysis, it has been possible to demonstrate a correlation between the local magnitudes of hydrostatic stress and tensile strain or octahedral stress and the appearance of specific tissue types within a fracture callus [2], [3]. A similar regulation mechanism using quantified limits for strain and hydrostatic pressure as stimuli for tissue differentiation has also been proposed [4]. An alternative theory suggests that tissue differentiation is regulated by a combined stimulus of octahedral shear strain and relative fluid velocity [5]. This model has been shown capable of predicting tissue differentiation during multiple regenerative events such as fracture healing [6], [7], osteochondral defect repair [8], [9], vertebral fracture repair [10], distraction osteogenesis [11]–[13], bone chamber ingrowth [14] and neoarthrosis formation [15], [16], providing strong corroboration for this hypothesis. In spite of this, understanding the relative importance and predictive ability of various biophysical cues as regulators of stem cell fate is challenging. For example, consideration of only a single mechanical stimulus such as deviatoric strain, volumetric strain or principal strain can lead to reasonably valid predictions of tissue differentiation during fracture repair [6], [17].

An inherent assumption of such hypotheses is that these mechanical signals act directly on mesenchymal stem cells (MSCs) to regulate their differentiation pathway. In conjunction, or perhaps alternatively, the local mechanical environment could also act indirectly to regulate MSC differentiation by inhibiting angiogenesis and hence the supply of oxygen and other factors to the wound site. Such inhibition of angiogenesis can lead to the development of hypoxic regions within a regenerating tissue, which may repress some differentiation pathways while promoting others. *In vitro* studies have shown severe impairment of adipogenesis and osteogenesis at low oxygen tensions [18]–[21], and a number of *in vivo* and *in silico* studies have highlighted the importance of angiogenesis for normal bone repair [22]–[28]. On the other hand, chondrogenesis is enhanced under hypoxic conditions [21], [29]–[32]. Furthermore, it has been found that cartilage formation is increased in more hypoxic fractures [33]. In addition to oxygen, other environmental cues are known to play a key role in regulating stem cell fate. It has been demonstrated that substrate stiffness directs stem cell differentiation [34]–[36]. Soft matrices that mimic the microenvironmental elasticity of brain tissue were shown to be neurogenic, stiffer matrices that mimic muscle tissue were found to be myogenic, while more rigid matrices that mimic collagenous bone were demonstrated to be osteogenic [34], [35].

The objective of this study was to test a new hypothesis for how environmental factors regulate stem cell differentiation during regenerative events such as fracture repair. Rather than assuming mechanical signals act directly on stem cells to determine their differentiation pathway, it was postulated that they act indirectly to regulate angiogenesis and hence partially determine the local oxygen environment within a regenerating tissue. Therefore, within the permissive environment of a fracture callus, which consists of a multitude of growth factors and cytokines, we hypothesized that it is the stiffness of the adjacent substrate and the local oxygen tension that determines the differentiation pathway of MSCs that invade a fracture callus. This hypothesis has been motivated by *in vitro* observations reported in the literature of how these factors in isolation regulate stem cell differentiation, and will be tested by attempting to simulate the spatial and temporal formation of different tissue types during fracture healing using a computational model based on this hypothesis.

## Methods

### Model of Stem Cell Differentiation

**Figure 1:**
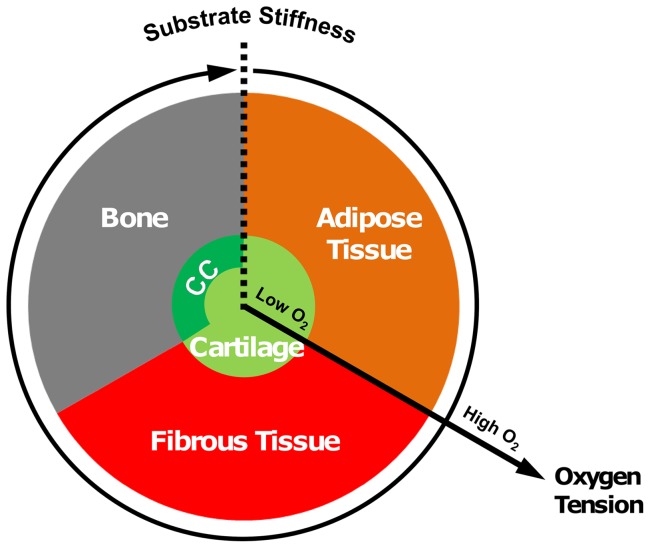
Tissue differentiation regulated by substrate stiffness and oxygen tension. The oxygen tension axis extends radially from the centre of the circle, low oxygen tension in the centre of the circle increasing towards the periphery. The substrate stiffness axis extends circumferentially in a clockwise direction from the right side of the dotted line at the top of the circle. The presence of a blood supply is also a prerequisite for formation of bone and marrow. (CC: Calcified Cartilage).

The prominent tissue types involved in fracture repair are cartilage, marrow (which in the medullary cavity of long bones consists primarily of fatty yellow marrow), bone and fibrous connective tissue [23], [37]. For this study, we have developed an algorithm whereby stem cell differentiation along either a chondrogenic, osteogenic or adipogenic lineage is regulated by the stiffness of the local substrate and the local oxygen tension (Fig. 1). The algorithm predictions can be described as follows:

1. **Chondrogenesis:** in regions of hypoxia.
2. **Osteogenesis:** in regions with sufficient oxygen that are adjacent to bony tissue (i.e. a stiff substrate).
3. **Adipogenesis:** in regions with sufficient oxygen that are adjacent to marrow (i.e. a soft substrate).
4. **Fibrogenesis:** in all other regions.

It has been shown that both osteogenesis and adipogenesis are repressed under hypoxic conditions [17]–[19]. It has also been shown that chondrogenesis is promoted under hypoxia [28]–[31]. Therefore, chondrogenesis is predicted by the algorithm when the local oxygen tension drops below a threshold value (*O*_2_^cartilage^), and is assumed to occur independent of the local substrate stiffness. The relationship between bone formation and blood supply has long been recognized [38], [39]. Blood vessels transport oxygen, nutrients and biological factors necessary for osteogenesis [38]. A similar relationship exists between blood supply and adipose tissue formation [40]–[42]. The presence of functional vascular supply is therefore a requirement for both adipogenesis and osteogenesis in this model. Should a sufficient blood supply, and hence oxygen supply, be present at a given point in the callus, the local substrate stiffness stimulus governs stem cell fate. *In vitro* studies have found that a stiff substrate can promote osteogenic differentiation while a soft substrate can promote adipogenic differentiation [34], [36]. Given that adipose tissue is a key component of marrow, the algorithm predicts adipogenesis and the reestablishment of the marrow cavity when the stiffness of the substrate is low. A stiff substrate leads to the prediction of osteogenesis and the formation of bone. Should any of these conditions not be met, fibrogenesis and fibrous tissue formation is predicted (Fig. 1).

The substrate stiffness stimulus at any point in the model is dependent upon the phenotype of surrounding elements (In this case ‘element’ refers to the discretized regions that make up the finite element model of the fractured bone and callus. This finite element model is used to predict the mechanical environment within the callus). Engler *et al* [34] refer to MSC differentiation regulated by the “elasticity of the microenvironment” of the cell. For example, the stiffness of osteoid matrix produced by osteoblasts is approximately 30 kPa, however, the stiffness of woven bone itself is orders of magnitude higher (in the order of GPa). In this tissue differentiation model, osteogenesis occurs when stem cells are adjacent to newly formed bone and hence in contact with osteoid as a substrate (and similarly for adipogenesis). In this implementation, specific threshold values of stiffness are therefore not required. It takes time for an element to “fill” with a newly forming tissue (e.g. bone). This is accounted for in a tissue formation rate, *TFR*, which simulates the progression of “an osteogenic front” across the element (larger elements take longer to fill). This limiting rate is defined in units of volume (of new tissue formed) per surface area of suitable substrate (stiff or soft) available. Cells at the very edge of the element “sense” the required substrate, differentiate and produce “osteoid” (in the case of bone). Next, cells slightly further away from the edge of the element would sense the substrate. Therefore, it takes time for this bone front to cross an element and the next element cannot sense the osteogenic substrate until the adjoining element is full of bone. How “full” an element is of bone is recorded and carried forward from iteration to iteration. A number of subiterations (per day) are carried out so that this calculation is accurate. The incorporation of this *TFR* allows the model to be independent of element size.

During endochondral ossification, hypertrophic chondrocytes are more prominent in close proximity to bone [43], [44]. Hypertrophic chondrocytes are assumed to direct the mineralisation of their surrounding matrix [45]. This calcification stiffens the cartilage tissue [46]. In this algorithm, cartilage becomes stiffened (by a factor of two) within close proximity to bone as a means to model this process of calcification. This calcified cartilage can be replaced by bone (endochondral ossification) assuming it can become adequately vascularized. Finally, during the remodelling phase of healing, bone resorption plays an important role in restoring the metabolic efficiency of the site by removing unnecessary bone. It has long been assumed that this resorption is strain related [47], [48]. Bone resorption occurs in this model when the deviatoric strain in a bone element drops below a threshold value (γ^resorption^) (Table 1). Mature bone is predicted following ten days of immature bone being predicted.

**Table 1:** Model parameters.

Model Parameter	Symbol	Source	Unit	Value
*Angiogenesis Diffusion Coefficient	*H*	Estimated	mm^2^/day	0.5
*Strain Threshold for Angiogenic Inhibition	*γ* ^angio^	[51]	%	6
Oxygen Diffusion Coefficient	*G*	[54]	m^2^/s	2.2E-09
Oxygen Consumption Rate	*Q*	[53]	fmol/cell/hr	98
Maximum Cell Density	*n* ^max^	[26], [70]	cells/mm^3^	5E03
Initial Oxygen Tension	*O*_2_^initial^	[55]	mmHg	74.1
*Maximum Tissue Formation Rate	*TFR*	Estimated	mm^3^/mm^2^/day	1
Bone Resorption Strain Limit	*γ* ^resorption^	[6]	%	0.005
Oxygen Tension Limit for Cartilage	*O*_2_^cartilage^	[18], [19]	%	3

* The effect of varying these parameters was investigated (see Results Section).

### Model of Angiogenesis

**Figure 2:**
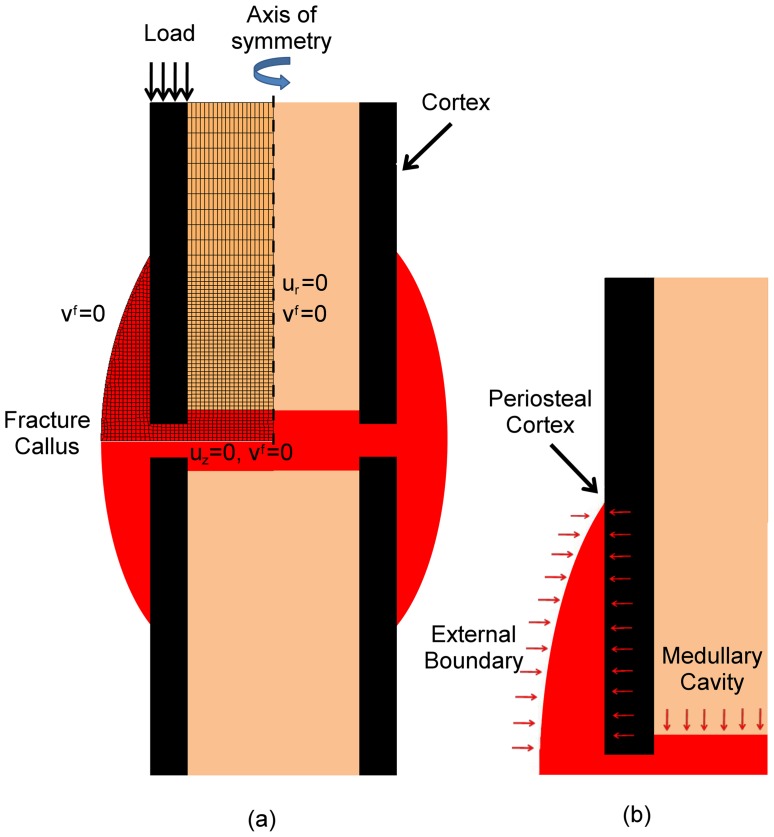
Finite element, cell and angiogenic models. (a): Finite element model with loading and boundary conditions. (b): boundary conditions for angiogenic and cell models. Radial displacement, axial displacement and fluid velocity are shown as *u_r_*, *u_z_*, and *v^f^* respectively.

Angiogenesis was modelled as a diffusive process [25]:
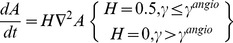
(1)Where *H* is the angiogenic diffusion coefficient (which represents the speed at which new blood vessels progress through the callus), *γ* ^angio^ is the threshold value of deviatoric strain for inhibition of blood vessel progression and *A* is the blood vessel concentration (Table 1). Blood vessels were assumed to invade the callus from the medullary cavity [49] and the periosteal cortex [50] (Fig. 2b). It is not clear in the literature whether vessels can also sprout from the lifted periosteum and surrounding muscle tissue (i.e. the external boundary of Fig. 2b). Simulations were performed with and without this boundary condition in order to investigate its effect on healing patterns. Points in the model where it is assumed blood vessels originated were assigned a blood vessel concentration of 100%. Any element with greater than 90% concentration was considered as having a blood supply present. The presence of a blood supply is necessary for osteogenesis or adipogenesis. Angiogenic progression was inhibited in regions of high deviatoric strain (γ). For the baseline simulation, we took 6% deviatoric strain as this threshold value (*γ* ^angio^), similar to previous computational models described in the literature [51]. However, given the uncertainty associated with this value, a parameter variation study was undertaken on its effect. As the rate at which blood vessels sprout and progress through a callus (represented by the diffusion coefficient, *H*, governing angiogenic progression), the tissue formation rate, *TFR,* and the threshold value of deviatoric strain for inhibition of blood vessel progression, *γ* ^angio^, have not been measured experimentally, the effect of changing these parameters was systematically investigated (see Results Section).

### Model of Oxygen Transport

Oxygen, *O*_2_, transport was described as a second diffusive process, the boundary conditions of which are dependent upon the state of the blood supply defined from the angiogenic model. Should an element have a blood supply present, the nodes of the element are assigned a fixed boundary condition equivalent to the initial or maximum oxygen concentration (see below). Oxygen consumption was considered a function of the local cell density [52]:
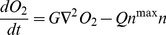
(2)Where *G* is the oxygen diffusion coefficient, *Q* is the cellular oxygen consumption rate, *O_2_* is the current oxygen tension, *n* is the cell concentration (ranging from a minimum of 0 to a maximum of 1) and *n* ^max^ is the maximum cell density. The local oxygen concentration at any point in the model is therefore dependent upon the initial oxygen state of the callus, the oxygen consumption rate (which in turn is dependent upon the local cell density) and proximity to a blood supply. MSC oxygen consumption rates vary within a range of values depending on a number of factors [53]. For simplicity, the consumption rate, *Q*, was assumed to be constant. The oxygen diffusion coefficient, *G*, was taken to be that of oxygen in blood [54]. The initial oxygen concentration (*O_2_* ^initial^) was taken to be 74.1mmHg [55] throughout the callus. The cell concentration, *n*, was obtained from the cell migration and proliferation model (see Appendix S1). Maximum cell density (*n* ^max^) was an order of magnitude estimate similar to the range of values used elsewhere [26]. Values of all model parameters are available from Table 1. With these values the predicted temporal changes in oxygen tension are presented and compared with experimental measurements in the results section.

### Finite Element Model

A finite element model was created similar to that used by Lacroix and Prendergast (2002) to predict the biophysical stimuli within a fractured long bone (Fig. 2a). Axisymmetric geometry and biphasic material properties were assumed (Table 2). The cylindrical bone diaphysis had an internal diameter of 14 mm, an external diameter of 20 mm and an external callus diameter of 28 mm. The fracture gap modelled spanned 3 mm. A 300N axial ramp loading of 0.5s was applied at the top of the cortical shaft to simulate weight bearing on the fractured bone. The callus is assumed to be initially filled with granulation tissue. The described model was implemented into the commercial finite element software package MSC Marc (version 2008r1, MSC Software Corporation, Santa Ana (CA), USA).

**Table 2:** Material properties.

Material Property	Granulation Tissue	Fibrous Tissue	Cartilage*	Marrow	Immature Bone	Mature Bone	Cortical Bone
Young's Modulus (MPa)	0.2^a^	2^b^	10^a^	2^a^	1,000^a^	6,000^c^	20,000^c^
Permeability (mm2)	1E-11^a^	1E-11^b^	5E-15^d^	1E-14^a^	1E-13^a^	3.7E-13^e^	1E-17^f^
Poisson's Ratio	0.167^a^	0.167^a^	0.167^a^	0.167^a^	0.3^a^	0.3^a^	0.3^a^
Fluid Dynamic Viscosity (Ns/m2)	1E-9	1E-9	1E-9	1E-9	1E-9	1E-9	1E-9
Porosity	0.8^a^	0.8^a^	0.8^a^	0.8^a^	0.8^a^	0.8^a^	0.04^g^

**a.** Lacroix and Prendergast (2002) [7]; **b.** Hori and Lewis (1982) [75]; **c.** Claes and Heigele (1999) [4]; **d.** Armstrong and Mow (1982) [76]; **e.** Ochoa and Hillberry (1992) [77]; **f.** Cowin 1999 [78]; **g.** Schaffler and Burr (1988) [79]. * Calcified cartilage was assumed to have a Young's Modulus of 20 MPa. All other properties are identical to cartilage.

### Iterative Procedure

**Figure 3:**
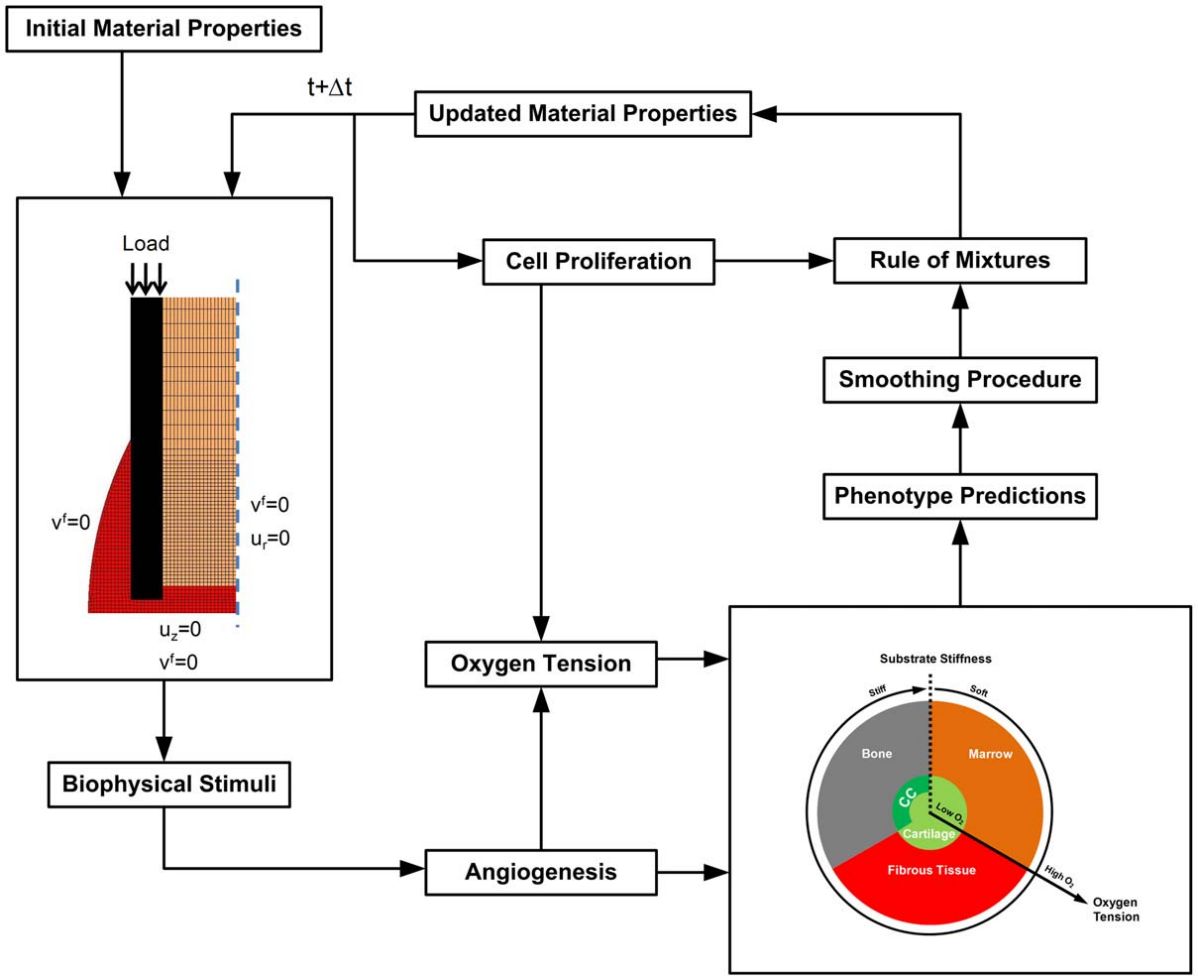
Iterative procedure for tissue differentiation hypothesis testing.

Tissue differentiation within the fracture callus was simulated via an iterative procedure similar to that described previously in the literature [7], [56] (Fig. 3). Within each iteration, a prediction of mechanical stimuli, cell density, substrate stiffness, blood supply and oxygen tension is made in order to enable the local phenotype to be determined based on the tissue differentiation algorithm. Firstly, a finite element model of a fracture callus is used to predict the spatial patterns of mechanical stimuli within the callus (see Finite Element Model Section). These mechanical stimuli influence angiogenic progression, which is inhibited in regions of high deviatoric strain. The oxygen tension is then dependent upon the initial oxygen environment, local blood supply, cell consumption rate and cell density (see Oxygen Transport Section). Local phenotype predictions are then made according to the tissue differentiation algorithm (Fig. 1). Cell proliferation and migration are modelled as a diffusive process (see Appendix S1) [56]. Tissue material properties are influenced by cell density according to the rule of mixtures as previously described (see Appendix S2) [7], [56], [57]. A temporal smoothing procedure is implemented to account for the delay between stimuli first acting on a cell and change of tissue type (see Appendix S3) [7], [56], [57]. Updated material properties are fed back into the finite element model for the next iteration of the cycle. This iterative process is continued until a solution is converged upon.

For the purposes of comparison, the same iterative procedure was implemented with stem cell differentiation regulated by a well established algorithm in which stem cell fate is dictated by a combined stimulus of octahedral shear strain and fluid velocity [58]. When this stimulus is high, fibrous tissue is predicted, when the stimulus is moderate, cartilaginous tissue is predicted and a low stimulus results in the prediction of bone formation. Bone resorption is predicted when the stimulus falls below a very low threshold level.

## Results

**Figure 4:**
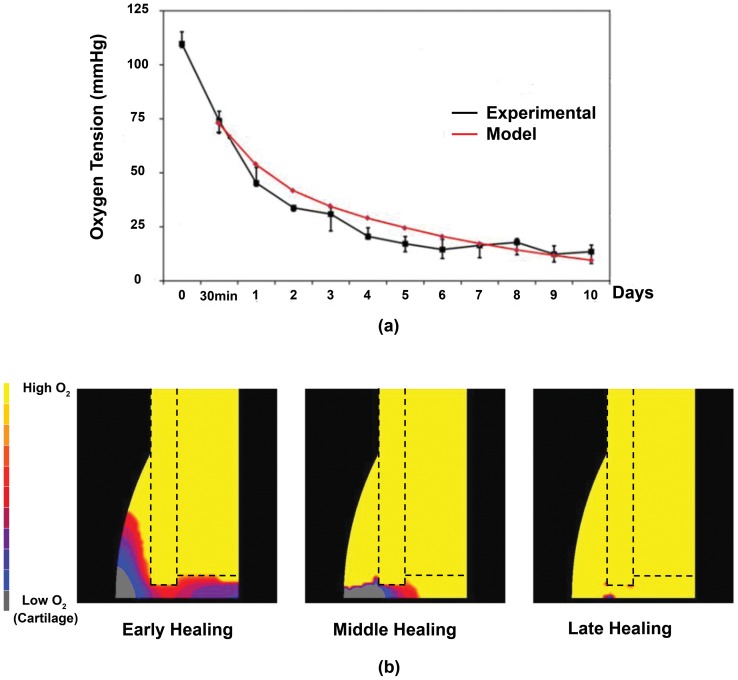
Oxygen model predictions. (a): Model predictions compared to experimental data for oxygen tension readings in the periosteal callus adjacent to the fracture gap (Image adapted from Epari et al (2008) with permission). (b): Predictions of oxygen tension in the callus at early, middle and late Stages of healing.

**Figure 5:**
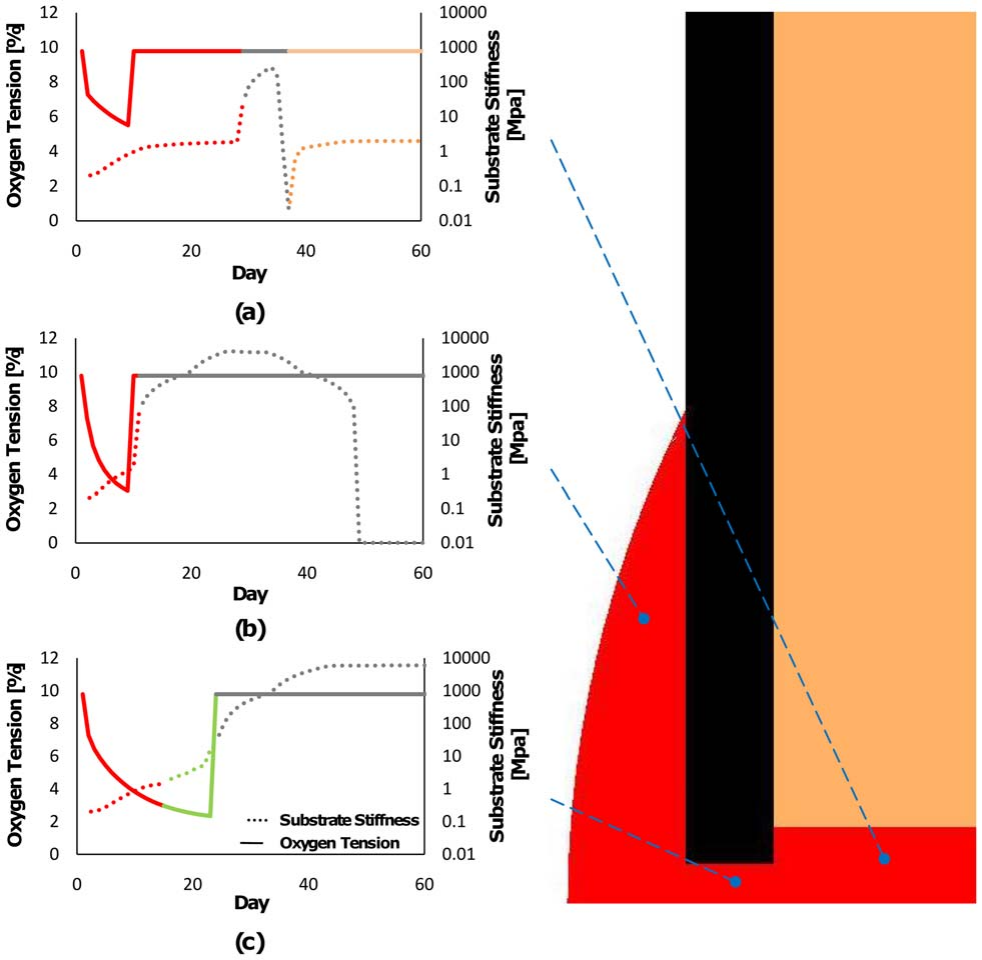
Model predictions for substrate stiffness and oxygen tension . Locations chosen as characteristic of the periosteal callus, fracture gap and endosteal callus respectively. It should be noted that substrate stiffness here refers to the macroscale stiffness of the regenerating tissue, where it is noted (as discussed in the manuscript) that the elasticity of the microenvironment of the cell is most likely different.

**Figure 6:**
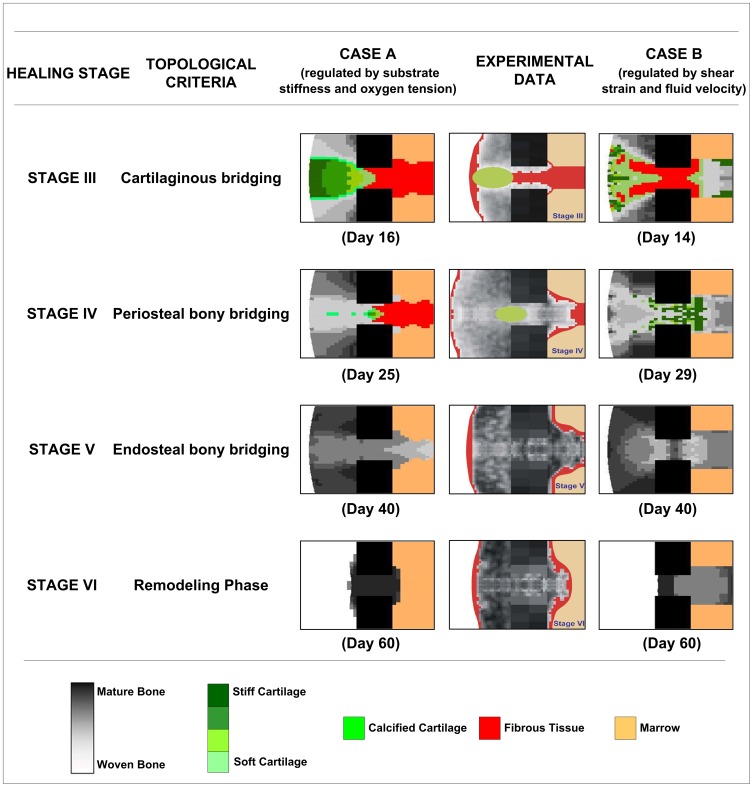
Model predictions versus experimental data. Model A: Model predictions for Stages III to VI of fracture healing when tissue differentiation is regulated by substrate stiffness and oxygen tension. Model B: Model predictions for Stages III to VI of fracture healing when tissue differentiation is regulated tissue shear strain and relative fluid velocity [6], [7]. Experimental Data: Averaged histological images obtained from an extensive study of fracture healing in sheep (Images adapted from Vetter et al (2010) with permission).

Model predictions of oxygen tension for the first ten days are compared to experimental measurements of oxygen tension in the periosteal region adjacent to the fracture gap [55] (Fig. 4a), demonstrating reasonably good agreement between the two. The temporal values of both oxygen tension and substrate stiffness vary throughout the facture callus, with different magnitudes predicted in the periosteal callus, fracture gap and endosteal callus (Fig. 5). In the endosteal callus (Fig. 5a), oxygen tension initially decreases but is restored to normal levels upon being vascularised early in the repair process. Fibrous tissue is initially predicted to form within this region of the callus (Fig. 5 and Fig. 6), hence the local substrate stiffness remains low. Eventually bone is predicted to form within the endosteal callus, initially at the stiff endosteal side of the cortex, and the substrate stiffness in this region of the model increases. This bone is eventually resorbed and replaced by marrow which progresses from the undamaged marrow body, resulting in a decrease in stiffness. In the fracture gap (Fig. 5c), the oxygen tension is predicted to decrease during the early stages of repair due to cellular consumption. Chondrogenesis proceeds, after initial fibrous tissue formation, followed by cartilage calcification, vascularisation and an increase in oxygen tension. In the upper periosteal callus (Fig. 5b), early vascularisation restores oxygen levels following initial decreases. Substrate stiffness in this region rapidly increases as bone, which is initially predicted to form on the stiff cortex, progresses into the lower cartilaginous periosteal callus. The hypoxic region that forms in the periosteal callus adjacent to the fracture gap early in healing later shifts towards the fracture gap (Fig. 4b). Cartilaginous tissue formation stabilises the callus and vascularisation eventually restores normal oxygen tension levels in the middle and latter stages of regeneration (Fig. 4b).

Predicted patterns of tissue differentiation were compared for the two models. In model A it is assumed that stem cell differentiation is regulated by substrate stiffness and oxygen tension, whereas in model B, differentiation is regulated by octahedral shear strain and relative fluid velocity [58]. The simulation results from both algorithms were compared to averaged histological images [37] for Stages III to VI of fracture healing (Fig. 6). Both models predict the major events of fracture repair. Bone formation originates in the upper periosteal callus and proceeds towards the fracture gap (Stages I and II). Cartilage forms in the periosteal region adjacent the fracture gap to form a cartilaginous bridge (Stage III). This cartilage undergoes endochondral ossification and leads to periosteal bony bridging (Stage IV). Endosteal bony bridging follows and the fracture gap is now completely bone (Stage V). Periosteal bone is then remodelled to restore the original bone structure (Stage VI). Some differences between the two models are noticeable upon closer examination of the model predictions.

For Stage III, model A (substrate stiffness and oxygen tension) predicts cartilaginous bridging of the periosteal callus and also less mature cartilage formation in the fracture gap. Bone formation has progressed from the upper callus to fill the periosteal region above the fracture gap. Some marrow formation is evident endosteally at the existing marrow body. The remainder of the internal callus remains as fibrous tissue. Model B (shear strain and fluid flow) predicts similar bone formation in the upper periosteal callus, cartilage formation in the outer periosteal callus and fibrous tissue in and adjacent to the fracture gap. Bone and cartilage are predicted to fill the remainder to the endosteal callus. For Stage IV, model A predicts bony bridging in the periosteal callus adjacent to the fracture gap. Some bone formation is predicted to originate from the endosteal cortex. The endosteal callus remains predominantly fibrous tissue. Model B also predicts bony bridging in the outer periosteal callus. The fracture gap and adjacent regions are predominantly cartilage with some bony regions also. The remainder of the endosteal callus is full of bone. For Stage V, both algorithms predict bone in the periosteal callus, endosteal callus and fracture gap. For Stage VI, model A predicts the resorption of the periosteal callus which was previously bone. The endosteal callus is also fully resorbed of bone which allows the recanalization and full restoration of the marrow of the medullary cavity. Model B also predicts full resorption of the periosteal callus but the endosteal callus remains full of bone.

**Figure 7:**
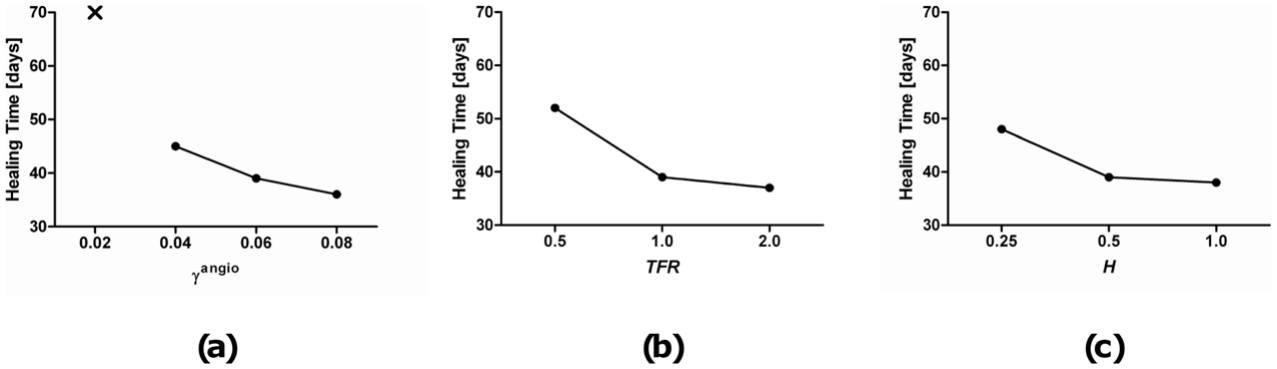
Effect on healing Time of parameter variations. (a): Healing time versus angiogenic strain threshold, *γ* ^angio^ (***X*** signifies the prediction of non-union) (b): Healing time versus tissue formation rate, *TFR*. (c): Healing time versus angiogenic diffusion coefficient, *H*.

A sensitivity analysis was performed to investigate the effect of modifying the angiogenic diffusion coefficient, *H*, the angiogenic strain threshold, *γ* ^angio^, and the tissue formation rate (*TFR*) (see Fig. 7). Models with the angiogenic threshold value increased to 8% deviatoric strain predicted slightly less cartilage and more bone formation in the early stages of healing in comparison to the baseline simulation. Models with the threshold value reduced to 4% deviatoric strain displayed slightly more cartilage and less bone formation in the early stages of healing in comparison to the baseline simulation. Healing, which we define as when the fracture gap is full of bone, occurred earlier for an increased angiogenic inhibition threshold (8%) and occurred later for a decreased angiogenic inhibition threshold (4%) (3 days earlier and 6 days later respectively, see Fig. 7c). Decreasing the threshold for angiogenic inhibition to 2% deviatoric strain resulted in a prediction of non-union (see Fig. 7a). Halving the angiogenic diffusion coefficient (0.25) resulted in slower healing (9 days later). Doubling the angiogenic diffusion coefficient (1.0) was predicted to only decrease the healing time by 1 day over the baseline simulation. Further increases in this coefficient appear to cause simulations to converge upon a minimum healing time and has no additional effect. In this case, the bone formation rate becomes the limiting factor. Increasing the bone formation rate, while the angiogenic diffusion coefficient remains constant, had a similar convergence upon a minimum healing time (see Fig. 7b).

The addition of a third angiogenic boundary condition to simulate a blood vessel source from surrounding soft tissues resulted in a slightly faster healing time (one day less) but again led to a similar spatial pattern of healing. The removal of the angiogenic source from the marrow cavity resulted in no healing from the endosteal side until very late in healing and an increased healing time (7 days later). If both periosteal and external boundary blood supply sources are not included in the simulation, healing fails to occur with the marrow cavity as the sole angiogenic source. Further simulations (data not shown) demonstrated that increasing the applied load from 300N to values over 700N also leads to predictions of fracture non-union.

## Discussion

A number of different hypotheses have been proposed for how extrinsic mechanical signals govern stem cell fate [3]–[5]. Support for these hypotheses has been provided by demonstrating that computational models based on such hypotheses can successfully predict aspects of tissue differentiation during regenerative events such as fracture healing. Despite *in vitro* studies identifying alternative environmental cues, such as substrate stiffness and oxygen tension, as regulators of stem cell fate, little is known about what role these cues play in regulating tissue differentiation during regenerative events *in vivo*. In this study, we demonstrate for the first time that the major events that occur during fracture healing can be predicted by a model that assumes substrate stiffness and oxygen tension regulate stem cell differentiation. In this model mechanical factors act indirectly to regulate stem cell fate by regulating angiogenesis and hence, in combination with cellular oxygen consumption, the local oxygen tension. The model predictions provide equally compelling data in support for this new hypothesis as previous studies [2], [4], [6], [7], [59] proposing that extrinsic mechanical signals act directly on stem cells to regulate their differentiation pathway.

A number of differences were observed between the model predictions based on substrate stiffness and oxygen tension (model A) and that of shear strain and fluid flow (model B) [5]. Endosteal cartilage is predicted by model B at both Stages III and IV of healing. This is due to high levels of fluid velocity contributing to the mechanical stimulus in the endosteal callus. Endosteal cartilage is not always observed histologically [37], and is not predicted by model A. In this model, oxygen tension in the endosteal callus does not decrease sufficiently to induce cartilage formation as there is a close angiogenic supply which progresses from the existing marrow of the medullary cavity. Model B (shear strain and fluid flow) predicts endosteal bone formation originating from the centre of the medullary cavity. A low mechanical stimulus leads to this prediction. Observations from histological images typically show that endosteal bone tends to originate from the endosteal cortex and progress towards the centre of the cavity [4], [37]. This experimental observation is captured in model A, due to the requirement of a high substrate stiffness for osteogenesis. In addition, a high fluid velocity stimulus prevents bone resorption of the endosteal callus in model B. Resorption regulated by strain alone allows this endosteal bone to be resorbed in model A. This permits the subsequent recanalization and restoration of the marrow of the medullary cavity as occurs *in vivo* [37]. It should be noted that more recent models of tissue differentiation regulated by shear strain and fluid velocity also predict resorption of the endosteal callus [60].

In developing this model, a number of questions related to the endochondral pathway were considered. The underlying mechanism behind cartilage calcification and endochondral ossification is not fully understood but it is believed that hypertrophic chondrocytes play a role in directing cartilage mineralization [45]. What drives chondrocytes to become hypertrophic in the first place is not something that is fully understood. Proximity to a blood supply will undoubtedly increase oxygen levels in cartilage immediately adjacent to vascularised bone. The local oxygen tension is one factor which may regulate chondrocyte hypertrophy [29], [61], with emerging evidence suggesting that low oxygen conditions suppress the hypertrophic phenotype. In support of this, it has been demonstrated that chondrocyte maturation and subsequent bone formation is delayed by antiangiogenic treatment [62]. In the growing bone, a layer of hypertrophic chondrocytes is found in close proximity to existing bone [43], [44]. Calcified cartilage most commonly occurs at the interface with adjacent bone. Based on these observations it was assumed in this model that chondrocytes adjacent to bone undergo hypertrophy and direct the calcification of the surrounding cartilage tissue in this region. Other factors, such as mechanical cues [63]–[65], have also been shown to regulate chondrocyte hypertrophy. Future models will consider these potential regulators of chondrocyte hypertrophy.

In undertaking this approach, we have assumed that mineralization precedes vascularisation, stiffening the tissue before vessel ingrowth. This stiffening provides some protection to blood vessels from possible shearing due to high strains at the interface. Only in the case where the stability provided by the calcified cartilage is not sufficient to facilitate progression of vascularisation will the calcified cartilaginous tissue not be replaced by bone. The alternative, vascularisation of the cartilaginous template preceding mineralization, is also possible. Further investigation is required to attempt to answer this question more affirmatively.

**Figure 8:**
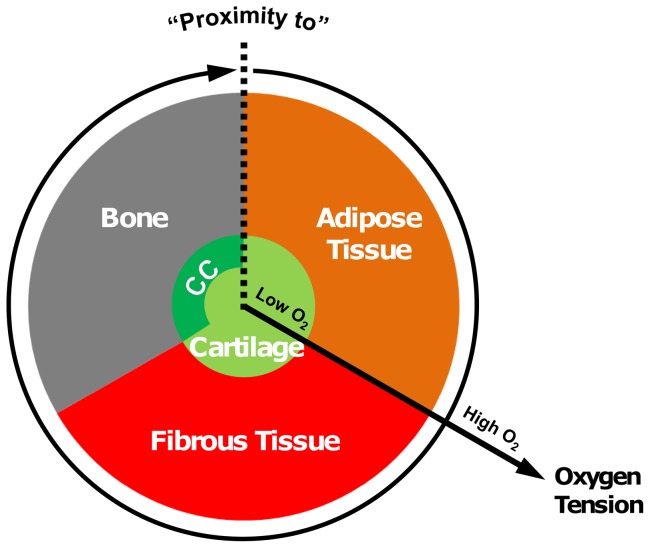
Tissue differentiation regulated by proximity and oxygen tension. The oxygen tension axis extends radially from the centre of the circle, low oxygen tension in the centre of the circle increasing towards the periphery. Bone and adipose tissue formation occur when there is sufficient oxygen tension “in proximity” to existing adipose tissue or bone fronts. The presence of a blood supply is also a prerequisite for formation of bone and adipose tissue. (CC: Calcified Cartilage).

The implementation of the substrate stiffness stimulus in this model could also be interpreted in a slightly different way, where the driving stimuli are oxygen tension and ‘proximity to’ bone (for osteogenesis) and ‘proximity to’ adipose tissue (for adipogenesis) (see Fig. 8). For example, in the presence of a blood supply (and hence sufficient oxygen), the osteogenic stimulus is pre-existing bone. One key factor associated with ‘proximity’ to a tissue is the local substrate stiffness or elasticity which it provides a cell and which is clearly a key environmental factor regulating MSC fate decisions. However, we also recognise that tissues themselves may be a source of growth factors, and hence proximity to a bone, for example, may also mean proximity to higher concentrations of growth factors or other biochemical cues necessary for osteogenesis. In either interpretation, the implementation is as performed in this study.

The model does have some limitations. Firstly, an axisymmetric geometry is adopted. It is assumed that the axisymmetric model will still provide a reasonable prediction of the environment within such a callus. Only axial loading is considered, implying that the model is most representative of well fixed fractures. Cell proliferation/migration and angiogenesis, modelled as diffusive processes, are simplified representations of complex *in vivo* processes. More complex models have been implemented [14], [24], [26], [28], [60] but we do not believe that implementing such models would alter dramatically the model predictions and, hence, the corroboration of the underlying model hypothesis. Inhibition of angiogenesis in this study is assumed to be as a result of a high shear strain stimulus but there is also the possibility that lower concentrations of blood vessels demonstrated in unstabilised fractures [23], [66], [67] may simply be due to the formation of avascular tissue (i.e. cartilage) itself. The oxygen model implemented in this study assumes a constant cell oxygen consumption rate. It has been shown that this is not the case [53]. Convection of regulatory factors is not considered. Again, our objective in this study was not to create a perfect model of oxygen transport and consumption, but merely to implement a model with sufficient predictive ability to allow us to test our hypothesis. It should also be noted that a simplified model of bone marrow reestablishment has been implemented in this study. We recognize that the marrow of the medullary cavity of long bones contains not only the marrow stroma and adipose tissue predicted by our algorithm, but also hematopoietic and lymphatic cells. Again, these simplifications were implemented to enable us to test the hypothesis of the study without introducing additional complexity. A specific stimulus for fibrous tissue formation is not offered by this tissue differentiation model. There is evidence that a mechanical stimulus can induce fibrous tissue formation [3] and this warrants further investigation. Fracture callus growth and size are also key factors not considered in this study [68], [69]. It should be noted that gradients in growth factors that may also regulate tissue differentiation [26], [70] were not considered. It is acknowledged that the presence of such factors may be critical to initiate stem cell differentiation. For example, it has recently been demonstrated that even well vascularised bone defects may not fully regenerate, which has been associated with a decreased expression of key regulatory factors such as BMP-2 and BMP-4 [71]. Only by explicitly including such factors into the model (as demonstrated, for example, by Geris *el al* (2010) [72]) can complex cases of non-union be simulated. Finally the model does not consider a role for substrate stiffness in regulating chondrogenesis, rather assuming it is regulated entirely by oxygen availability which is most likely a simplification.

In spite of these limitations this model, which assumes that stem cell fate is regulated by substrate stiffness and oxygen tension, can successfully predict all the major events of fracture repair. In doing so we have demonstrated how disparate environmental cues, which *in vitro* have been shown to independently regulate stem cell fate, are potentially integrated by MSCs *in vivo* to drive differentiation during regenerative events such as fracture healing. Of course, the results of these simulations only provide preliminary support for the underlying model hypothesis, and should not be used to conclude one hypothesis (e.g. differentiation regulated by substrate stiffness and oxygen) is more valid than another (e.g. differentiation regulated by shear strain and fluid flow). All that can definitively be stated is that one cannot reject either hypothesis tested as part of this computational mechanobiological analysis. Furthermore, model predictions should not be used to support the idea that substrate stiffness and oxygen tension alone entirely determine stem cell differentiation. Other biochemical cues are also most likely required. If the fracture callus is viewed as a permissive environment, where multiple growth factors and cytokines are present that will allow MSCs to differentiate down multiple different pathways, this study provides support for the hypothesis that the oxygen tension and substrate stiffness play a key role in determining cell fate in such a permissive environment. Whether these factors act alone, or in combination with extrinsic biophysical signals such as hydrostatic pressure, strain and fluid flow, to regulate MSC differentiation is a critical question. Mounting experimental data from *in vitro* studies suggests all of these factors are important [73], [74]. Decoupling the relative importance of these various cues is challenging using computational models alone, but may in the future be possible by integrating computational models with appropriately designed *in vitro* and *in vivo* studies of stem cell differentiation. Future work in our lab will explore this key question, as well as exploring the role of substrate stiffness and oxygen tension in regulating stem cell differentiation during other regenerative events in the body.

## Supporting Information

Appendix S1 **Cell model.**
(DOCX)

Appendix S2 **Rule of mixtures.**
(DOCX)

Appendix S3 **Temporal smoothing procedure.**
(DOCX)
