# Supplementary material for: Substrate Stiffness and Oxygen as Regulators of Stem Cell Differentiation during Skeletal Tissue Regeneration: A Mechanobiological Model
Source: PLoS One. 2012 Jul 24;7(7):e40737. doi: 10.1371/journal.pone.0040737 (PMC3404068; doi:10.1371/journal.pone.0040737)
Supplement: Appendix S1 — Cell model. (DOCX) [file pone.0040737.s001.docx]

Appendix S1: Cell Model

MSCs both migrate [[74](#_ENREF_74)] and proliferate [[75](#_ENREF_75)] within the fracture callus. It was assumed that the net process of these effects can be modeled via a diffusion model [[7](#_ENREF_7)]:

$\frac{dn}{dt}=D\nabla^{2}n$ (A1)

where *D* is the stem cell diffusion coefficient and *n* is the current stem cell concentration. Stem cells originated from the marrow of the medullary cavity [[76](#_ENREF_76)], the inner cambial layer of the periosteum [[77](#_ENREF_77)] and from the surrounding muscle tissues. Before the influx of MSCs into the callus, the fracture callus is initially full of granulation tissue.
