# Supplementary material for: Substrate Stiffness and Oxygen as Regulators of Stem Cell Differentiation during Skeletal Tissue Regeneration: A Mechanobiological Model
Source: PLoS One. 2012 Jul 24;7(7):e40737. doi: 10.1371/journal.pone.0040737 (PMC3404068; doi:10.1371/journal.pone.0040737)
Supplement: Appendix S2 — Rule of mixtures. (DOCX) [file pone.0040737.s002.docx]

**Appendix S2 Rule of Mixtures**

The gradual change of an element from one tissue type to another is accounted for in the model of the element material properties. The material properties are calculated *via* the rule of mixtures:

$E=\frac{n^{\max}-n}{n^{\max}}E_{granulation}+\frac{n}{n^{\max}}E_{tissue}$ (A2)

The Young’s Modulus of an element, *E*, is calculated as a function of the element’s cell density (*n*), the maximum cell density (*n*^max^), the Young’s Modulus of granulation tissue (*E* granulation) and the Young’s Modulus of the tissue the element is differentiating towards. The rule of mixtures is applied to the other material properties in a similar manner.
