# Supplementary material for: Substrate Stiffness and Oxygen as Regulators of Stem Cell Differentiation during Skeletal Tissue Regeneration: A Mechanobiological Model
Source: PLoS One. 2012 Jul 24;7(7):e40737. doi: 10.1371/journal.pone.0040737 (PMC3404068; doi:10.1371/journal.pone.0040737)
Supplement: Appendix S3 — Temporal smoothing procedure. (DOCX) [file pone.0040737.s003.docx]

**Appendix S3: Temporal Smoothing Procedure**

A temporal smoothing procedure is included in the model in order to avoid any numerical instability that might occur as a result of rapid changes in material properties. An array of *k* values is defined and initially the properties of granulation tissue are inputted into this array. After the first iteration (*i* = 1), the material property of the predicted tissue phenotype is placed at the end of the array. All existing properties are moved along one position and the *i*^th^ value in the array is deleted. Should this be performed a second time the array would look like:

$E=\left[E_{granulation},E_{granulation},...,E_{i=1},E_{i=2}\right]$ (A3)

At the *k*^th^ iteration (*i* = *k)*:

$E=\left[E_{i=1},E_{i=2},...,E_{i=n-1},E_{i=n}\right]$ (A4)

The material properties for each iteration were calculated via:

$E=\frac{1}{k}\sum_{j=1}^{k} E_{j}$ (A5)

For this study *k* was taken to be 10. The other material properties are calculated in a similar manner.
